# Mindful leadership training for public administration executive staff: a non-randomized controlled trial

**DOI:** 10.1186/s40359-026-04493-7

**Published:** 2026-04-11

**Authors:** Susana Iris Rueda-Sánchez, Mayte Navarro-Gil, Alejandra Aguilar-Latorre, Alberto Barceló-Soler, Javier García-Campayo

**Affiliations:** 1 Department of Finance and Public Administration, Government of Aragon, Zaragoza, 50001 Spain; 2 Department of Medicine, Psychiatry and Dermatology, Faculty of Medicine, University of Zaragoza, Zaragoza, 50009 Spain; 3 Department of Psychology and Sociology, Faculty of Education, University of Zaragoza, Zaragoza, 50009 Spain; 4 Institute for Health Research Aragón (IIS Aragón), Zaragoza, 50009 Spain; 5 Research Network On Chronicity, Primary Care and Health Promotion (RICAPPS, RD24/0005/0004), Carlos III Health Institute, Madrid, 28029 Spain; 6 Department of Psychology and Sociology, Faculty of Human Sciences and Education of Huesca, University of Zaragoza, C/Valentín Carderera, 4, Huesca, 22003 Spain

**Keywords:** Mindful leadership, Transformational leadership, Work engagement, Well-being; public administration, Non-randomized controlled trial

## Abstract

**Background:**

Rapid societal and organizational changes pose challenges for public administrations, where leadership style influences employee functioning and organizational effectiveness. Mindful leadership integrates mindfulness-based self-regulation (present-moment awareness, attentional control, and non-reactivity) with leadership behaviors to support ethical, relational, and effective decision-making.

**Objective:**

To evaluate the effectiveness of a mindful leadership training program compared with an active control condition (management skills training) among executive staff in a public administration setting.

**Methods:**

We conducted a parallel non-randomized controlled trial in a public administration context. Executive staff were recruited via organizational invitation and snowball procedures and assigned to mindful leadership training (*n* = 41) or management skills training (*n* = 24). Leadership, work engagement, mindfulness-related skills, and well-being outcomes were assessed at baseline, immediately post-intervention, and at six-month follow-up. Group-by-time effects were tested using repeated-measures models (including the group × time interaction) and results are reported with corresponding effect estimates.

**Results:**

Group × time interactions were not statistically significant across outcomes. Descriptively, the mindful leadership group showed time-related improvements in work engagement, transformational leadership, and mental well-being indicators, whereas the management skills group showed lower scores at follow-up for these outcomes. Other measures showed no clear change over time.

**Conclusions:**

Mindful leadership training may be a feasible and potentially beneficial approach for supporting leadership-related and well-being outcomes among public administration executive staff. Given the non-randomized design and unequal group sizes, further randomized and adequately powered trials are needed.

**Trial registration:**

ClinicalTrials.gov Identifier: NCT07132931 (released 12 August 2025).

**Supplementary Information:**

The online version contains supplementary material available at 10.1186/s40359-026-04493-7.

## Introduction

The rapid changes experienced by society and organizations have their equivalent in public organizations. Elements such as workforce diversity, aging, technological transformation, or the evolution of services pose significant challenges for public administrations to undergo processes of change and modernization [16]. As stated in the Basic Statute of Public Employees (EBEP, by its acronym in Spanish) [6], public administrations "must adapt to the needs of our time in line with the reforms being undertaken in other European Union countries and in the European community itself."

In parallel, authors like Choudhary et al. [4] highlight the importance of leadership style, identifying how it influences employees' extra-role behavior, intra-role performance, and positive self-perception of work. Meanwhile, Rana et al. [18] link it to organizational commitment, team effectiveness, lower occupational stress, and better conflict management strategies.

Mindfulness-based approaches are grounded in contemplative science and self-regulation frameworks, emphasizing the training of attention and emotion regulation skills that can influence behavior in demanding contexts [3].

These capacities have been proposed as relevant for leadership functioning, particularly in complex interpersonal environments [13].

Mindful leadership refers to the application of mindfulness—intentional, present-moment awareness with an attitude of openness and non-judgment—to leadership processes and behaviors. Conceptually, mindful leadership emphasizes leaders’ capacity to sustain attention, increase self-awareness, and act with authenticity in complex interpersonal contexts, while promoting ethical and compassionate decision-making. Recent integrative work has proposed that mindful leadership can be understood through core capacities such as attention, awareness, and authenticity, which may translate into more attuned, values-consistent leadership behaviors [5].

From a self-regulation perspective, mindfulness practice may strengthen leaders’ attentional control and emotion regulation, reducing automatic reactivity under stress and facilitating more constructive responses to demanding work situations. In turn, these mechanisms are expected to support leadership effectiveness and relational outcomes, and ultimately contribute to employees’ engagement and well-being. Consistent with prior reviews of mindfulness in leadership contexts, the strongest evidence to date suggests beneficial associations with leader functioning and interpersonal outcomes, although the intervention literature remains heterogeneous and context-dependent [24].

Empirical studies of mindful leadership training have increased in recent years, mainly in sectors such as healthcare and education, with early trials and pilot studies reporting improvements in stress-related outcomes and professional functioning among leaders and professionals receiving mindful leadership courses. However, these studies have largely been conducted outside public administration, limiting their direct applicability to public-sector executive staff operating under distinct bureaucratic constraints and service-oriented demands [21].

The training in mindful leadership, proposed in this study, begins by supporting the person who leads and expecting a subsequent impact on the organization and the community at large. That is, it proposes learnings that are good for people, good for the organization, and good for the community. Thus, it is expected to contribute to the well-being and performance of those who lead and, by extension, to their teams of collaborators, and to the effectiveness in public service.

Despite growing interest in mindfulness-based approaches in organizational settings, empirical evidence on mindful leadership interventions within public administration remains scarce. Most studies have been conducted in private-sector organizations or in health/education contexts, often using observational designs or without active control conditions. Consequently, there is limited controlled evidence on whether mindful leadership training yields measurable improvements in leadership style, work engagement, and well-being among public-sector executive staff, a group operating under distinct bureaucratic and service-oriented demands.

We examined whether mindful leadership training, compared with an active management-skills training, was associated with more favorable trajectories over time (baseline, post-intervention, 6-month follow-up) in:

- H1: leadership-related outcomes (transformational leadership);
- H2: work engagement;
- H3: mindfulness-related skills (mindfulness facets and self-compassion) and well-being indicators.

Given the pragmatic non-randomized design, these hypotheses were treated as theory-informed and exploratory, and conclusions were framed accordingly.

Therefore, the main objective of this study was to analyze the effectiveness of the “mindful leadership” intervention, compared with an active control condition of “management skills” (standard leadership training in the specific public administration under study), on leadership, work engagement, mindfulness-related skills, and well-being outcomes among executive staff in a public administration setting. Outcomes were assessed at baseline, immediately post-intervention, and at a six-month follow-up.

## Methods

### Study design and setting

This study was a pragmatic parallel non-randomized controlled trial conducted in a public administration setting (Government of Aragón, Spain) between 2019 and 2023, with assessments at baseline (T0), immediate post-intervention (T1), and 6-month follow-up (T2).

### Participants and recruitment

Participants were executive staff employed by the Government of Aragón who voluntarily applied for leadership training offered by the Aragón Institute of Public Administration (IAAP). Eligibility criteria were defined by IAAP and included: (1) holding managerial positions (Service Chiefs and Section Chiefs; Groups A1–A2), (2) length of service (priority to longer tenure), (3) employment status (priority to permanent staff), and (4) number of training actions in the previous year (priority to fewer training actions). Applicants not meeting these criteria were not admitted. Training opportunities were publicly announced by IAAP (including registration deadlines), and all admitted participants were invited to participate in the research study. Written informed consent was obtained prior to data collection.

### Allocation (non-random assignment)

Group allocation was non-random and followed IAAP’s routine training administration (participants attended the training edition to which they were admitted). A non-probabilistic snowball approach was used only to increase participation in the research assessments among eligible staff and did not determine training admission or group allocation. Internal validity may therefore be affected by self-selection and non-random assignment; this is addressed through covariate adjustment and discussed as a limitation.

### Intervention

Firstly, the active control group received a 30-h training program in "management skills," consisting of six sessions, each lasting five hours. This training was conducted with a group of 24 participants. During the program the following contents were worked on: a) Concept of 360º evaluation on leadership skills and people management. b) My Role, key relationships. Required profile. Situational leadership. c) Styles of conduct in managing people. d) Management of teams and meetings. e) Conflict management. Management of emotions. f) Evaluation of the transfer to the job.

Secondly, the experimental group "mindful leadership" received a 25-h training program, consisting of 7 sessions, each lasting three hours, along with four hours of individual work. This training was conducted in two groups, with 43 participants in total (18 and 25 participants, respectively). During the program the following contents were worked on: a) Conscientious leadership in public administration. b) Lead from Emotional Intelligence. c) Lead from Mindfulness. d) Lead from acceptance. e) Lead from compassion. f) Positive interventions. g) Well-being of the leader. h) Follow-up session. Living a conscious leadership.

Both interventions are proposed with a high practical content and continuous interaction between teachers and participants. The following methodology is used:

- 1) Development of theoretical content through master classes. 2) Practical classes and exercises for experiential knowledge. 3) Resolution of doubts of participants. 4) Exchange of experiences between participants.

The duration and structure of each program were determined by IAAP as part of routine professional development offerings and reflected feasibility constraints (e.g., organizational scheduling and maximum class capacity). In particular, the management skills program was delivered in a single cohort with a maximum capacity of 24 attendees, whereas the mindful leadership program was delivered in two cohorts (18 and 25 participants) to accommodate demand and scheduling. Because the programs differed in format and total contact hours (30 h vs. 25 h + 4 h individual work), dose differences may partly account for observed patterns and are considered a limitation of the comparative interpretation.

### Procedure

The information on the courses and the information on the voluntary enrolment of the participants in the courses was published in the Official Gazette of Aragon (BOA, by its acronym in Spanish). In particular, the intervention of the control group was advertised in the general BOA (nº. 192; 2019, September 24), and the intervention of the intervention group was offered in the Aragonese Institute of Social Services (IASS, by its acronym in Spanish) (nº. ZA-0125/2021; 2021, October 15).

The sampling technique that was used was non-probabilistic snowball sampling (i.e., participants were asked to encourage others to participate) [11].

The study was conducted between the years 2019 and 2023. It involved a 7-week intervention consisting of 7 sessions (1 session per week) for the experimental group and a 6-week intervention consisting of 6 sessions (1 session per week) for the active control group.

A blinded research technician, unaware of participant assignments, collected data from participants using questionnaires administered at baseline (pre-intervention), immediately after the intervention (within a period of 2 to 7 days after the last intervention session) (post-intervention), and at a 6-month follow-up after the intervention completion. Data entry and coding of the collected data were performed by another blinded research technician. Another blinded research technician conducted outcome assessments and data analysis. All collected information was treated in accordance with the provisions of the current laws on personal data protection.

### Measurements

We collected sociodemographic information at baseline on age, gender (female or male), education (none, primary, secondary or university), current job performance time, work time in administration and days off work over the past 12 months.

Physical well-being and mental well-being (MW) were assessed using two single-item self-ratings included in an ad hoc sociodemographic questionnaire. Participants were asked: “Please rate your physical well-being according to the following scale: very poor (1), poor (2), fair (3), good (4), very good (5)” and “Please rate your mental well-being according to the following scale: very poor (1), poor (2), fair (3), good (4), very good (5)”. Higher scores indicate better perceived well-being.

#### Variables related to leadership

Leadership was assessed using the Transformational Leadership Questionnaire. This is assessed using the Rafferty and Griffin questionnaire [17], developed based on the Multifactor Leadership Questionnaire [2], and adapted into Spanish by Salanova et al. [20]. This instrument comprises 15 items grouped into five dimensions:

- Vision: Three items (alpha = 0.74) (e.g., "perfectly understands the group's objectives").
- Inspirational Communication: Three items (alpha = 0.88) (e.g., "says positive things about the department/unit").
- Intellectual Stimulation: Three items (alpha = 0.84) (e.g., "encourages thinking about old problems in new ways").
- Support: Three items (alpha = 0.93) (e.g., "considers the team members' personal needs").
- Recognition: Three items (alpha = 0.96) (e.g., "personally congratulates when excellent work is done").

The internal consistency of the TL in our sample at pre-intervention showed good values, with α = 0.82.

Work Engagement was assessed using the Work Engagement Questionnaire. This is assessed using 17 items from the Spanish version of the Utrecht Work Engagement Scale (UWES; [19]), which includes three dimensions:

- Vigor: Six items (alpha = 0.85) (e.g., "I can continue working for long periods of time").
- Dedication: Six items (alpha = 0.90) (e.g., "I am enthusiastic about my job").
- Absorption: Five items (alpha = 0.84) (e.g., "time flies when I am working").

The internal consistency of the UWES in our sample at pre-intervention showed excellent values, with α = 0.90.

#### Variables related to Mindfulness

Mechanistic outcomes include self-reported mindfulness skills, self-compassion, and experiential avoidance.

Mindfulness skills were assessed using the short version of the Five Facet Mindfulness Questionnaire (FFMQ; [1]), measured pre-post intervention. This abbreviated version of the FFMQ (FFMQ-15) was analyzed for structure and psychometric properties by Gu et al. [8]. It encompasses the same dimensions as the original FFMQ-39. The only difference lies in the inclusion of three items for each dimension or facet, resulting in the following: Observation (1, 6, and 11), Description (2, 7, and 12); Acting with Awareness (3, 8, and 13); Non-judging (4, 9, and 14); and Non-reactivity to Experience (5, 10, and 15). Furthermore, Gu et al. [8] found that the short version of FFMQ-15 demonstrates a consistent factorial structure akin to the long version FFMQ-39, with both versions exhibiting correlations among facets. This leads to the conclusion that both versions possess psychometric properties suitable for assessing the variable of mindfulness [8]. The internal consistency of the FFMQ-15 in our sample at pre-intervention showed questionable values, with α = 0.68.

Self-compassion was assessed using the Spanish version of the Self-Compassion Scale-Short Form (SCS-SF; [7]). This 12-item questionnaire assesses how respondents perceive their actions toward themselves during difficult times and measures the following six components of self-compassion (with reverse coding of negative aspects): self-kindness, self-judgment, common humanity, isolation, mindfulness, and over-identification. Each item is rated on a scale of 1 to 5 (*“almost never”* to *“almost always”*). The total score is the sum of the direct and reverse-scored items and ranges between 12 and 60, with higher scores associated with greater levels of self-compassion. The internal consistency of the SCS-SF in our sample at pre-intervention was good, with α = 0.86.

#### Variables related to well-being

Well-being was assessed using the Pemberton Happiness Index (PHI) Scale [9]: The PHI scale consists of two sections. The first section evaluates remembered well-being and includes 11 items related to different domains of remembered well-being: general well-being (items 1 and 2), eudaimonic well-being (items 3 to 8), hedonic well-being (items 9 and 10, with item 10 being reverse-scored), and social well-being (item 11). Participants respond to statements such as "I am very satisfied with my life" or "I feel very close to the people around me" by indicating their level of agreement or disagreement on a scale from 0 to 10, where 0 represents "Completely Disagree" and 10 represents "Completely Agree." The second section consists of 10 items related to the well-being experienced the previous day. These include 5 positive experiences (e.g., "I had a good time with someone") and 5 negative experiences (e.g., "I was worried about personal matters"). Scores obtained from both sections can be transformed into a single well-being index.

The internal consistency of the PHI in our sample at pre-intervention showed good values, with α = 0.81.

As outcomes were assessed through self-report questionnaires, we implemented procedural remedies to reduce potential common method bias, including standardized administration, assurance of confidentiality, and temporal separation of measurements across three time points (baseline, post-intervention, and 6-month follow-up), with blinded research staff collecting and processing the data. In addition, we selected instruments that assess conceptually distinct constructs (leadership, work engagement, mindfulness-related skills, and well-being), which supports construct distinctiveness and reduces the likelihood that results are solely attributable to measurement overlap.

### Ethics

Ethics approval was granted by the Research Ethics Committee of the Aragon (PI21/244; 30–06–2021). The study was developed in accordance with the Helsinki Declaration and its updates. All subjects provided informed consent before group allocation. All collected data was processed as stipulated in the current Spanish legislation regarding the protection of personal data (Law 3/18, Dec 5th). Once the data was collected it was anonymized for data analysis and only used for the study purposes.

This study was reported following CONSORT guidance; a completed CONSORT checklist is provided as a supplementary file.

### Statistical analysis

Descriptive analysis (frequencies and percentages for categorical variables; means and standard deviation for continuous variables), and univariate analysis (independent samples t-test and Chi-squared test) was used to examine between-group differences in sociodemographic and psychological data between the two groups in each measurement. General Linear Modelling (repeated-measures ANOVA) was performed to examine group, time, and the group × time interaction effects on study outcomes.

As a measure of effect size, Partial Eta Squared (η_p_^2^) was utilized, and variables showing baseline differences were controlled for in the analyses. The significance level was set at 0.05 using two-sided tests. The analyses followed a complete case approach using IBM SPSS Statistics software (version 25.0) [10].

Given the pragmatic, non-randomized nature of the study and the fixed cohort sizes determined by the training editions, we conducted a post hoc sensitivity analysis to estimate the minimum detectable effect size (MDES).

No additional statistical tests for common method variance (e.g., Harman’s single-factor test or CFA marker approaches) were conducted; therefore, common method bias cannot be fully ruled out.

Using a two-sided α = 0.05 and the observed group sizes (*n* = 41 and *n* = 24), the study had 80% power to detect a standardized between-group difference of approximately Cohen’s d = 0.73. Therefore, the available sample was sufficiently powered to detect moderate-to-large between-group effects, whereas smaller effects may not have been detectable and should be examined in adequately powered randomized trials.

## Results

**Fig. 1:**
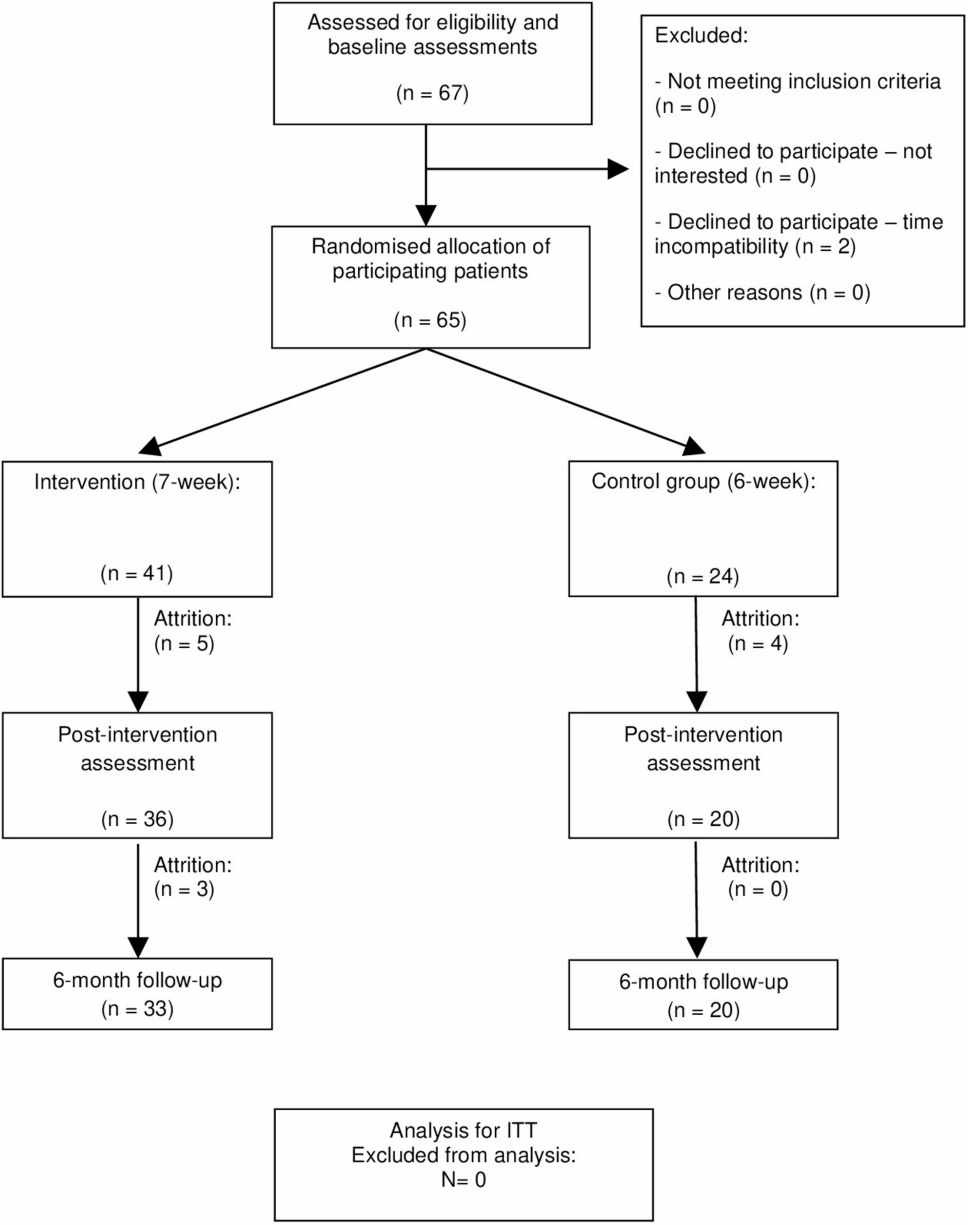
Flowchart of the study: allocation, sampling and monitoring of the participants

At baseline, 41 participants started in the 7-week intervention group, and 24 participants started in the 6-week control group. By the 6-month follow-up, 33 participants remained in the intervention group, and 20 participants remained in the control group (Fig. 1).

Table 1 summarizes baseline demographic, occupational, and psychological characteristics by group. Significant baseline between-group differences were observed for gender, physical well-being, mental wellness, FFMQ, SCS-SF, and PHI (Table 1). The majority of participants were women aged over 51, having accumulated more than 15 years of experience in Administration, and holding positions ranging from 1 to 5 years in their current roles.

**Table 1:** Sociodemographic and psychological characteristics of participants at baseline

Variables	Control (*n* = 24)	Intervention (*n* = 41)	*p*
Gender*, woman n (%)*	11 (45.8)	36 (87.8)	**0.001**
Age*, n (%)*			
*18 to 30 y/o*	1 (4.2)	-	0.182
*31 to 40 y/o*	6 (25)	4 (9.8)	
*41 to 50 y/o*	8 (33.3)	15 (36.6)	
*older than 51 y/o*	9 (37.5)	22 (53.7)	
Working time in the Administration*, n (%)*			
*1 to 5 years*	2 (8.3)	4 (9.8)	0.224
*6 to 10 years*	1 (4.2)	4 (9.8)	
*11 to 15 years*	7 (29.2)	4 (9.8)	
*more than 15 years*	14 (58.3)	29 (70.7)	
Time spent in your current position*, n (%)*			
*Less than 1 year*	2 (8.3)	9 (22)	0.211
*1 to 5 years*	15 (62.5)	16 (39)	
*5 to 10 years*	3 (12.5)	10 (24.4)	
*More than 10 years*	4 (16.7)	6 (14.6)	
Days off work last year, *M* (*SD*)	6.08 (19.15)	16.32 (59.53)	0.418
Physical well-being, *M* (*SD*)	4.33 (0.63)	3.88 (0.45)	**0.04**
Mental Wellness, *M* (*SD*)	4.29 (0.55)	3.59 (0.59)	**< 0.001**
TL, *M* (*SD*)	66.91 (7.89)	65.12 (9.09)	0.424
UWES, *M* (*SD*)	75.5 (16.53)	74.09 (12.55)	0.701
FFMQ, *M* (*SD*)	51.04 (5.42)	46.65 (5.85)	**0.004**
SCS-SF, *M* (*SD*)	39.17 (8.03)	35.09 (7.35)	**0.044**
PHI, *M* (*SD*)	96.58 (9.42)	90.68 (11.47)	**0.037**

*UWES* Utrecht Work Engagement Scale *FFMQ* Five Facet Mindfulness Questionnaire, *SCS-SF* Self-Compassion Scale-Short Form, *PHI* Pemberton Happiness Index, *TL* Multifactor Leadership Questionnaire

*p*: *p*-value for independent samples t-test or Chi-squared test. Bold values denote statistical significance at the *p* < 0.05 level

**Fig. 2:**
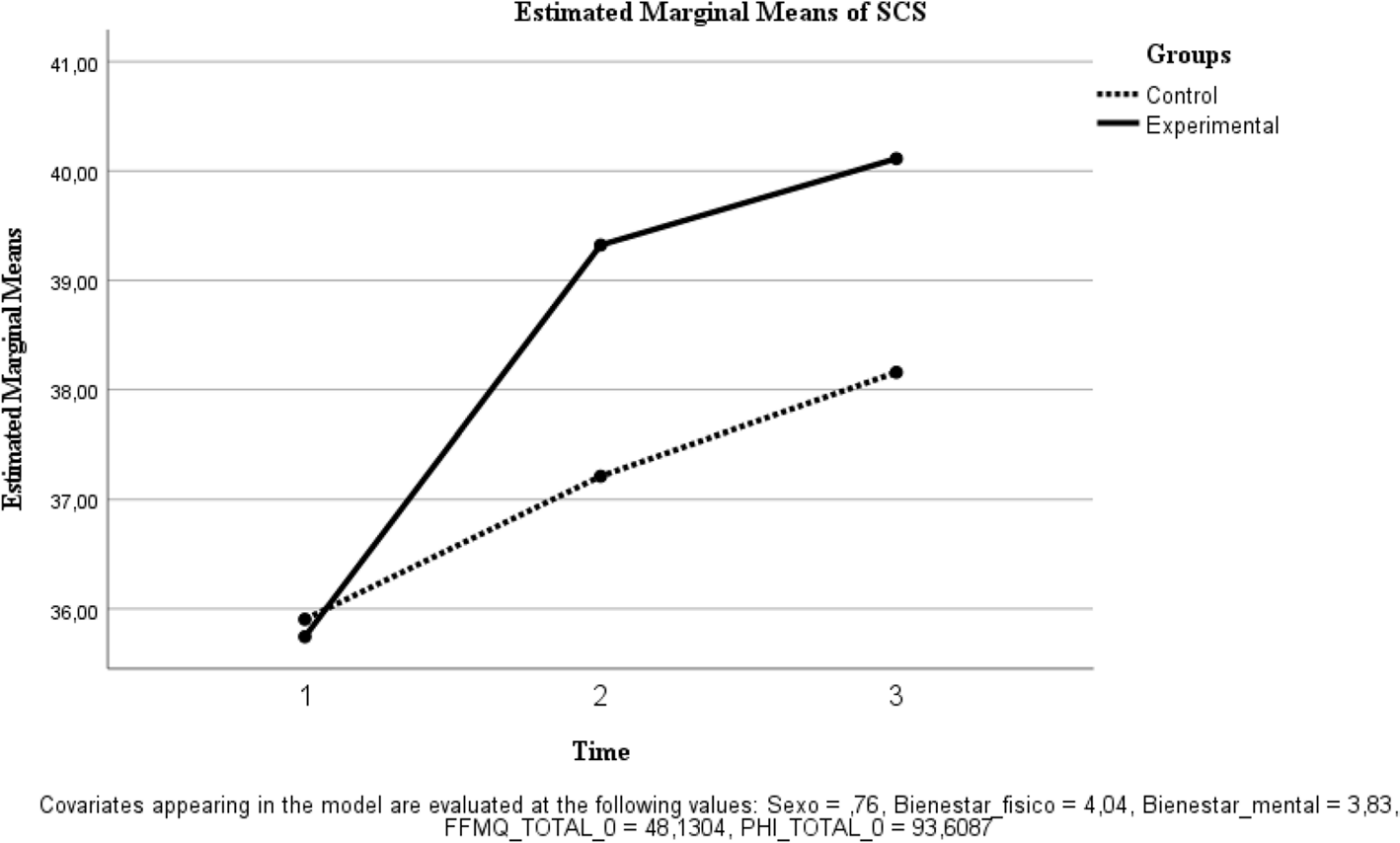
Estimated Marginal Means of SCS

**Fig. 3:**
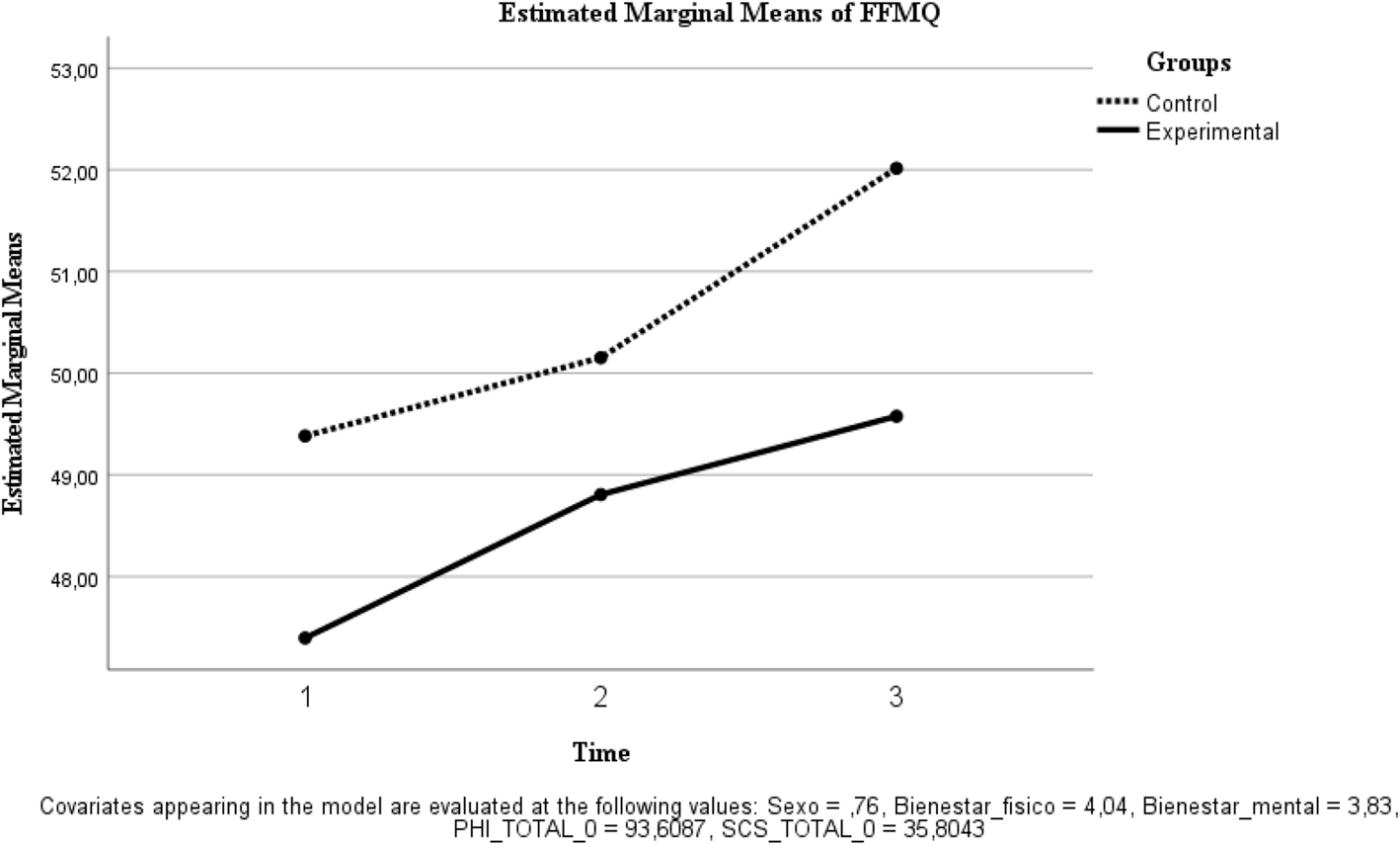
Estimated Marginal Means of FFMQ

**Fig. 4:**
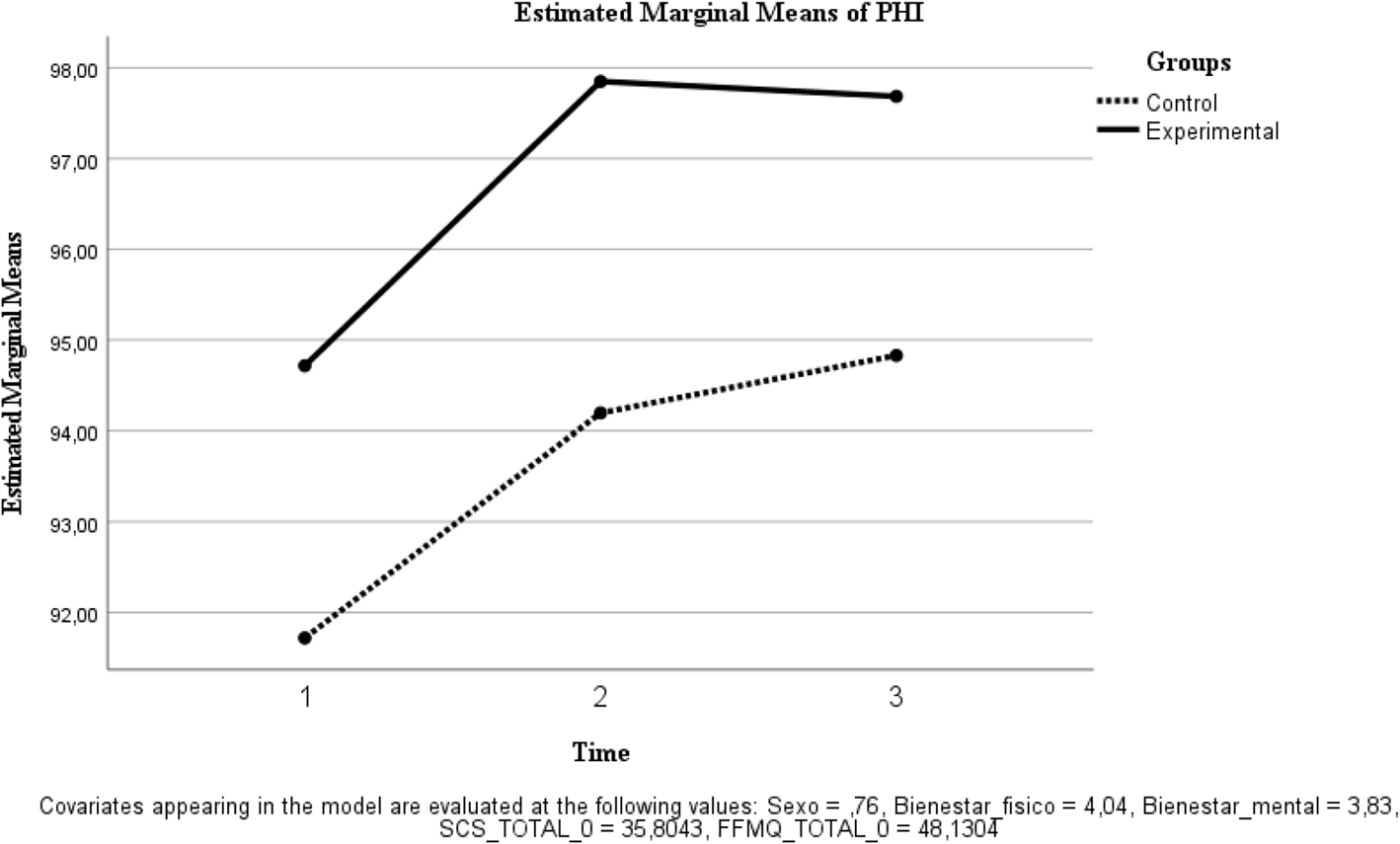
Estimated Marginal Means of PHI

**Fig. 5:**
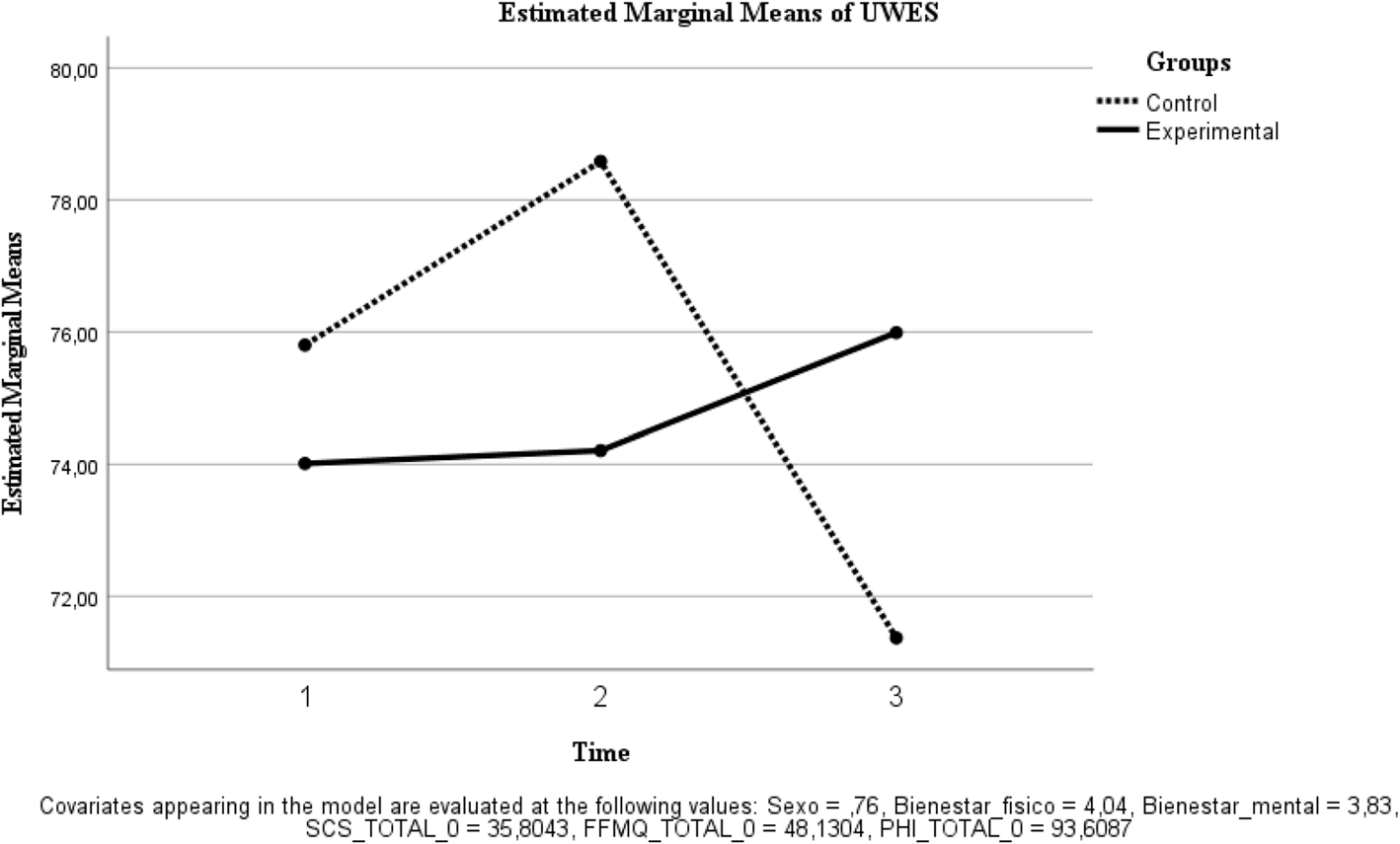
Estimated Marginal Means of UWES

**Fig. 6:**
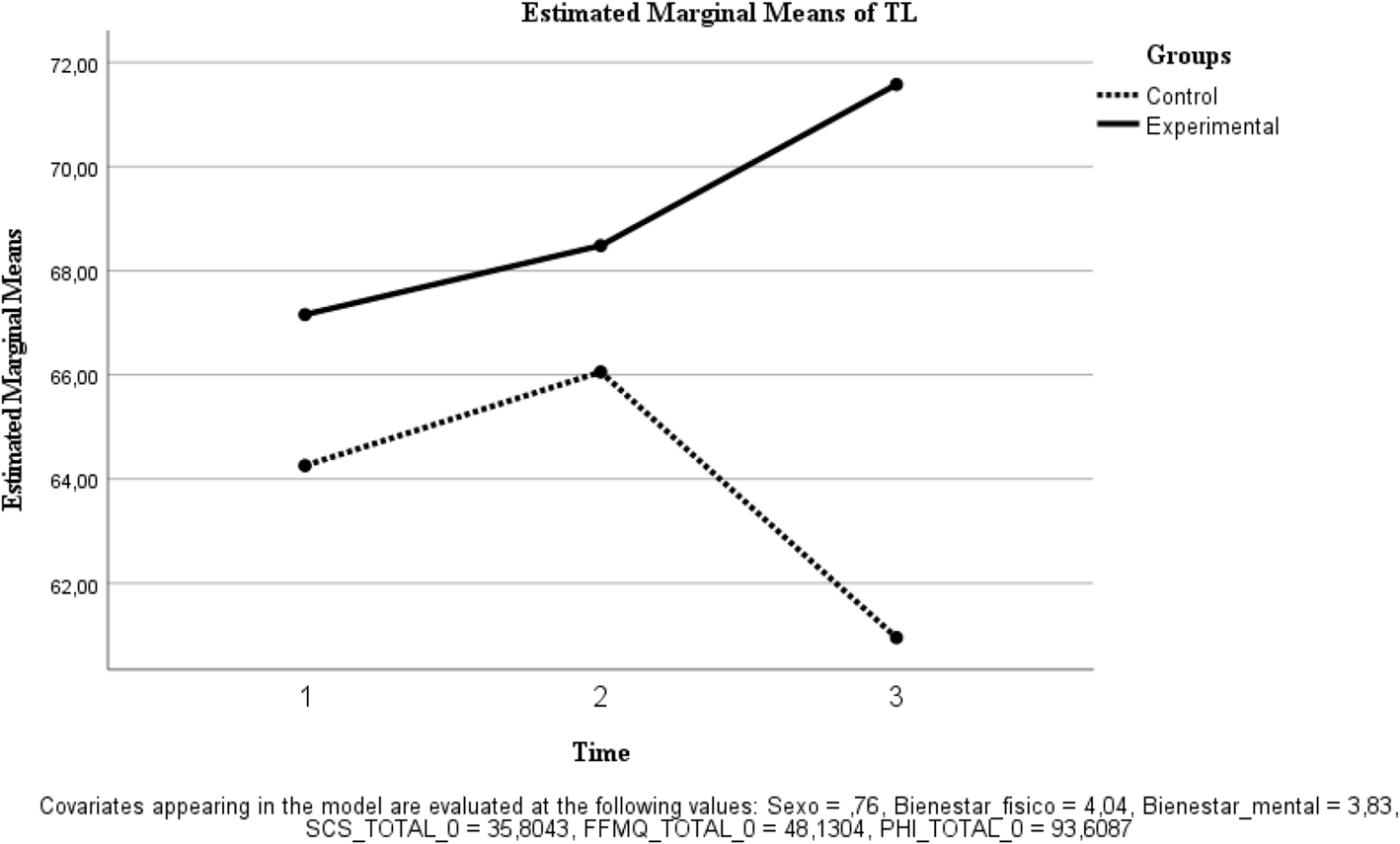
Estimated Marginal Means of TL

**Fig. 7:**
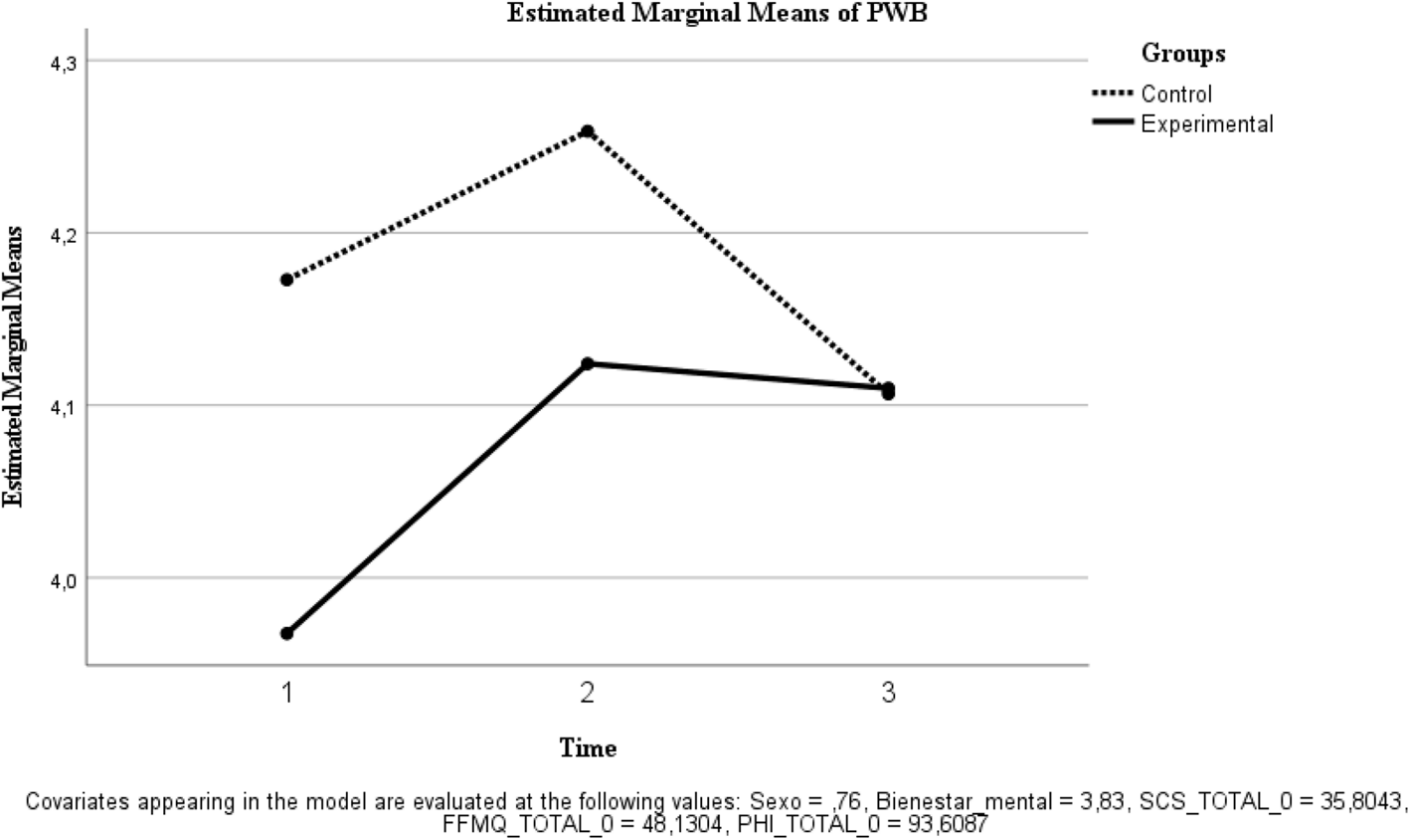
Estimated Marginal Means of physical well-being

**Fig. 8:**
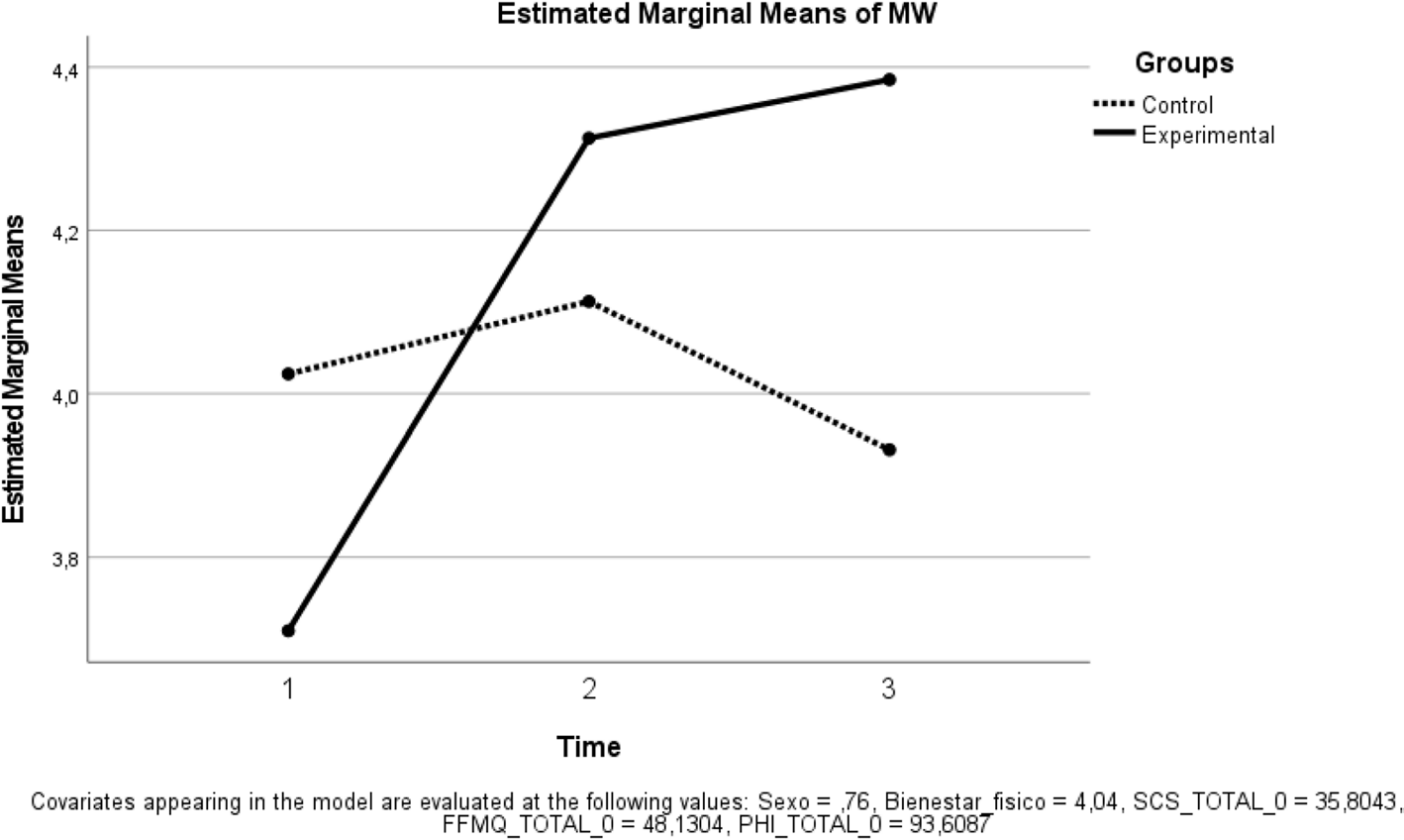
Estimated Marginal Means of Mental Wellness

Table 2 reports descriptive statistics for each outcome by group at baseline (T0), post-intervention (T1), and 6-month follow-up (T2), as well as exploratory (unadjusted) between-group comparisons of change scores. Primary longitudinal inference is based on repeated-measures ANOVA effects (time and group × time interaction), with estimated marginal means shown in Figs. 2, 3, 4, 5, 6, 7 and 8. Given that group × time interactions were not statistically significant, descriptive trajectories are reported cautiously and without implying differential effectiveness.

**Table 2:** Outcome variables of each group in each measurement

**Variables**	Control	Intervention	Exploratory between-group comparison of change scores (unadjusted independent t-test)
Physical well-being, *M* (*SD*)			
T0	4.33 (.637)	3.88 (.458)	
T1	4.35 (.489)	4.07 (.594)	
T2	4.15 (.489)	4.06 (.443)	
T1-T0	.00 (.458)	.08 (.500)	.541
T2-T0	-.25 (.444)	.18 (.391)	<.001
Mental Wellness, *M* (*SD*)			
T0	4.29 (.550)	3.59 (.59)	
T1	4.20 (.523)	3.93 (.594)	
T2	4.06 (.429)	4.00 (.459)	
T1-T0	-.05 (.394)	.61 (.728)	<.001
T2-T0	-.30 (.571)	.66 (.777)	<.001
TL, *M* (*SD*)			
T0	66.91 (7.895)	65.12 (9.094)	
T1	67.85 (7.058)	68.30 (8.892)	
T2	63 (10.146)	70.42 (9.663)	
T1-T0	2.40 (6.908)	3.08 (7.710)	.743
T2-T0	−3.50 (9.150)	3.72 (7.989)	.004
UWES, *M* (*SD*)			
T0	75.5 (16.539)	74.09 (12.553)	
T1	76.5 (14.358)	75.19 (12.049)	
T2	71.20 (15.866)	76.24 (13.432)	
T1-T0	2.65 (7.421)	1.36 (8.149)	.561
T2-T0	−3.60 (7.549)	1 (8.170)	.046
FFMQ, *M* (*SD*)			
T0	51.04 (5.425)	46.65 (5.85)	
T1	51.85 (5.142)	48.16 (4.513)	
T2	52.90 (4.8)	47.81 (7.273)	
T1-T0	.70 (4.268)	1.75 (4.094)	.369
T2-T0	1.70 (5.079)	1.18 (6.993)	.774
SCS-SF, *M* (*SD*)			
T0	39.17 (8.037)	35.09 (7.385)	
T1	40.25 (7.966)	38 (5.104)	
T2	39.550 (8.293)	38.87 (6.474)	
T1-T0	.75 (3.446)	3.13 (4.350)	.039
T2-T0	.78 (5.452)	4.75 (5.505)	.015
PHI, *M* (*SD*)			
T0	96.58 (9.426)	90.68 (11.474)	
T1	99.95 (10.485)	94.33 (11.022)	
T2	99.95 (8.876)	94.81 (12.266)	
T1-T0	2.05 (5.462)	3.61 (8.445)	.461
T2-T0	2.90 (5.485)	4.03 (9.531)	.631

Between-group comparisons of change scores (T1–T0; T2–T0) are exploratory and unadjusted for multiple testing. Therefore, *p*-values should be interpreted cautiously and are provided for descriptive purposes only. Primary longitudinal inference is based on repeated-measures ANOVA effects (time and group × time interaction) reported in the Results and illustrated by estimated marginal means (Figs. 2, 3, 4, 5, 6, 7 and 8). Abbreviations: TL, Transformational Leadership; UWES, Utrecht Work Engagement Scale; FFMQ, Five Facet Mindfulness Questionnaire; SCS-SF, Self-Compassion Scale-Short Form; PHI, Pemberton Happiness Index

Self-compassion (SCS-SF). No significant time effect was observed [F(1, 39) = 0.906, *p* = 0.347; ηp^2^ = 0.023], and the group × time interaction was not significant [F(1, 39) = 0.357, *p* = 0.553; ηp^2^ = 0.009] (Fig. 2).

Mindfulness skills (FFMQ-15). No significant time effect was observed [F(1, 39) = 0.035, *p* = 0.853; ηp^2^ = 0.001], and the group × time interaction was not significant [F(1, 39) = 1.525, *p* = 0.224; ηp^2^ = 0.038] (Fig. 3).

Well-being (PHI). No significant time effect was observed [F(1, 39) = 0.002, *p* = 0.968; ηp^2^ < 0.001], and the group × time interaction was not significant [F(1, 39) = 0.995, *p* = 0.325; ηp^2^ = 0.025] (Fig. 4).

Work engagement (UWES). A significant time effect was observed [F(1, 38) = 4.798, *p* = 0.035; ηp^2^ = 0.112], whereas the group × time interaction was not significant [F(1, 38) = 0.009, *p* = 0.926; ηp^2^ < 0.001] (Fig. 5).

Transformational leadership (TL). A significant time effect was observed [F(1, 38) = 4.818, *p* = 0.034; ηp^2^ = 0.113], whereas the group × time interaction was not significant [F(1, 38) = 2.491, *p* = 0.123; ηp^2^ = 0.062] (Fig. 6).

Physical well-being. No significant time effect was observed [F(1, 39) = 1.677, *p* = 0.203; ηp^2^ = 0.041], and the group × time interaction was not significant [F(1, 39) = 0.743, *p* = 0.394; ηp^2^ = 0.019] (Fig. 7).

Mental wellness (MW). A significant time effect was observed [F(1, 39) = 7.593, *p* = 0.009; ηp^2^ = 0.163], whereas the group × time interaction was not significant [F(1, 39) = 0.398, *p* = 0.532; ηp^2^ = 0.010] (Fig. 8).

## Discussion

In this study, we aimed to assess the effectiveness of a mindful leadership intervention compared with an active control condition (management skills training) among executive staff in a public administration setting, focusing on leadership, engagement, mindfulness-related skills, and well-being outcomes.

Overall, the primary longitudinal analyses did not show statistically significant group × time interactions for the study outcomes. Accordingly, the study does not support causal claims or differential effectiveness between programs; the findings should be interpreted as preliminary and hypothesis-generating. For self-compassion (SCS) [15], mindfulness skills (FFMQ) [1], global well-being (PHI) [9], and physical well-being, we did not observe significant changes over time nor evidence of differential trajectories between groups. This pattern is consistent with broader workplace mindfulness evidence indicating that effects on work-related outcomes can be heterogeneous and sometimes modest, particularly in applied settings and across different outcome domains [14, 23, 25].

For work engagement (UWES) [22], transformational leadership (TL), and mental wellness (MW), significant time effects were observed. Descriptively, UWES, TL, and MW scores tended to improve across time points in the mindful leadership group, whereas the control group showed lower scores at follow-up for some outcomes,however, group × time interactions were not statistically significant, indicating that differential effects between programs were not clearly supported by the primary inferential tests. Prior workplace mindfulness reviews and meta-analyses generally report beneficial effects on well-being and some work-related outcomes, but also highlight variability across studies and limited evidence for certain performance indicators [12, 14, 25]. These findings are also broadly aligned with leadership-focused evidence suggesting potential links between mindfulness and leadership-related functioning, while emphasizing the need for more controlled intervention research in leadership contexts [5, 24].

These findings should be interpreted in light of the study design. Because the trial was non-randomized and baseline differences were present for several variables, observed changes may partially reflect self-selection, contextual factors, or regression to the mean despite statistical control. In addition, the reliance on self-report outcomes may have increased susceptibility to social desirability and shared method variance. Together with the modest sample size and unequal groups, these factors may have limited the ability to detect small differential effects between programs—an issue frequently noted in the broader workplace mindfulness and leadership mindfulness literature [14, 24].

## Theoretical implications

These findings add to the emerging literature on mindful leadership by providing evidence from an under-studied context: public administration executive staff. While the primary longitudinal tests did not show clear differential effects between programs, the observed time-related changes in engagement, leadership, and perceived mental wellness are compatible with theoretical accounts linking mindfulness-based training with self-regulation capacities that may be relevant for leadership functioning in complex public-sector environments.

## Practical implications

From an applied perspective, this study shows the feasibility of implementing structured leadership-development programs within routine public administration training. Mindful leadership training may be considered as a potential complement to standard management-skills programs by incorporating practice and reflection components that are acceptable to executive staff. Embedding similar initiatives within public-sector training structures may support leadership development and workplace well-being, although further research is needed to confirm comparative effectiveness.

## Strengths and limitations

This study has several strengths. Firstly, it was conducted within the domain of public service, which remains relatively underexplored in research, particularly concerning public executive personnel. Secondly, the developed leadership development methodology can be replicated in other public organizations, offering a valuable contribution to the field. Additionally, the long-term follow-up provided insights into the sustainability of the intervention's effects. Moreover, the incorporation of performance evaluation measures such as engagement and transformational leadership adds depth to the evaluation of mindfulness and contemplative practices programs. Lastly, efforts were made to align mindfulness and contemplative sciences in the workplace context with their ethical foundations, aiming to humanize practitioners regardless of organizational impacts.

Despite these strengths, several limitations warrant consideration. Firstly, the study primarily focused on examining the effects of mindfulness and contemplative practices on individual leaders, neglecting potential impacts on their teams and collaborators. Secondly, the investigation did not delve into the factors contributing to mindfulness experiences and contemplative practices in the workplace, which could offer valuable insights for future research. Thirdly, the measured aspects might have been influenced by social desirability bias, highlighting the need for observing subsequent behavior. Future studies should incorporate multi-source outcomes (e.g., supervisor/peer ratings or objective indicators) and formal measurement models to further address common method variance.

In addition, as both programs were implemented as routine training offerings, they differed in structure and dose (7 weeks vs. 6 weeks; and total contact hours), and the intervention was not delivered through a fully manualized protocol with formal fidelity/adherence assessment. These factors may limit replicability and may confound comparative interpretation between groups. Expectancy and motivational biases could not be fully controlled in this real-world context and should be considered when interpreting the findings.

Moreover, physical well-being and mental wellness were assessed using single-item ad hoc measures, which may have limited psychometric precision compared with validated multi-item instruments. In addition, some measures showed only modest internal consistency in our sample (e.g., the FFMQ-15, α = 0.68). This level of reliability may increase measurement error and attenuate observed associations and intervention effects, potentially reducing the likelihood of detecting small true effects. Therefore, findings involving these measures should be interpreted with caution, and future studies should consider alternative indicators or complementary assessment methods. Furthermore, incorporating organizational strategies alongside individual practice may be necessary for achieving positive effects on well-being. Finally, the inclusion of non-practitioners or individuals with different perspectives in future research could help mitigate biases and enrich findings. Additionally, the non-randomized design may have introduced baseline imbalances and self-selection effects despite statistical control of baseline differences. The unequal and relatively small group sizes and the use of a complete-case approach may have limited statistical power, particularly for detecting small effects; this is consistent with the post hoc sensitivity/MDES analysis, and results should therefore be interpreted cautiously and confirmed in adequately powered randomized trials.

The intervention program (“conscious leadership”) was specifically developed for this study and was feasible to implement within the routine training context. Its duration and frequency were compatible with participants’ schedules and allowed time for practice between sessions. Future evaluations should incorporate standardized fidelity/adherence assessment and multi-source outcomes (e.g., supervisor/peer ratings or objective indicators) to strengthen interpretability and to better assess potential organizational implications.

## Future research directions

Future research should further explore the effectiveness of different leadership interventions in organizational settings and examine potential mechanisms through which mindful leadership and management-skills training may influence work engagement, transformational leadership, and mental wellness. Qualitative studies capturing participants’ experiences, along with quantitative assessments of mediators and moderators, would be valuable. Longitudinal studies with larger samples are also needed to evaluate the sustainability of changes and potential delayed effects. Additionally, examining variability across organizational contexts and cultural settings, as well as individual differences (e.g., prior mindfulness experience), may help identify for whom and under what conditions these programs are most beneficial. Future evaluations should prioritize stronger causal designs, such as cluster-randomized trials (e.g., randomizing departments/units), stepped-wedge designs embedded in routine training roll-out, or matched sampling/propensity-score approaches when randomization is not feasible. In addition, future studies should standardize intervention dose and incorporate fidelity/adherence assessment and multi-source outcomes.

## Conclusions

In conclusion, while this study provides insights into mindful leadership training in public administration, further research using randomized and adequately powered designs is needed to confirm these findings and clarify mechanisms and long-term organizational outcomes.

## Supplementary Information

Supplementary Material 1.

## Data Availability

The datasets used and/or analysed during the current study are available from the corresponding author on reasonable request.
